# Effect of the Anti-Inflammatory Diet in People with Diabetes and Pre-Diabetes: A Randomized Controlled Feeding Study

**DOI:** 10.14200/jrm.2019.0107

**Published:** 2019-02-15

**Authors:** Heather Zwickey, Angela Horgan, Doug Hanes, Heather Schiffke, Annie Moore, Helané Wahbeh, Julia Jordan, Lila Ojeda, Martha McMurry, Patricia Elmer, Jonathan Q Purnell

**Affiliations:** a Helfgott Research Institute, National University of Natural Medicine, Portland, OR, USA; b Oregon Health and Science University, Portland, OR, USA; c University of Colorado School of Medicine, Aurora, CO, USA

**Keywords:** Anti-inflammatory diet, pre-diabetic diet, cytokines and diet, weight loss

## Abstract

**Introduction:**

Inflammation underlies a variety of chronic medical conditions, including diabetes. The anti-inflammatory diet, one that excludes foods that may stimulate inflammation and includes foods that reduce inflammation, may improve inflammatory biomarkers in people with diabetes and pre-diabetes.

**Study Design:**

Thirty participants with diabetes or pre-diabetes were randomized (2:1) in a controlled feeding study that compared the anti-inflammatory diet (*n*=20) to a control diet (*n*=10) based on the American Diabetes Association recommendations. Diets were matched for protein, carbohydrate, fat, and fiber content as closely as possible. Participants were fed an isocaloric diet for 2 weeks, followed by continued *ad libitum* feeding in their dietary group assignment for an additional 4 weeks. All meals were prepared by the study team.

**Outcomes:**

Primary outcomes included inflammatory markers, including cytokines and hsCRP. Secondary outcomes included body weight and biomarkers for cardiovascular disease and diabetes.

**Results:**

Both diets resulted in trends in reduced markers of inflammation, especially with weight loss. In addition, glucose, lipids, and triglycerides all trended downward, also non-significantly and equally in both groups.

**Conclusion:**

Dietary change can improve inflammation as well as other cardiometabolic risk factors. In this study, the anti-inflammatory diet did not affect markers of inflammation more than the control diet.

## INTRODUCTION

It is becoming increasingly clear that there is a strong relationship between inflammation and obesity.^1–4^ One mechanism of this relationship involves adipokines and cytokines.^1^ Obese people have increased subcutaneous adipocytes (fat cells) that produce chronically elevated levels of leptin.^5^Higher leptin secretion has been linked with increased production of pro-inflammatory cytokines Tumor Necrosis Factor alpha (TN0046α), Interleukin 1 beta (IL-1β), and Interleukin 6 (IL-6).^3^ Increased levels of these cytokines and high-sensitivity C-reactive protein (hs-CRP) reflecting longstanding immune activation has been implicated in the pathogenesis of insulin resistance and chronic diseases such as type 2 diabetes.^6–8^ This suggests that inflammation accompanying obesity may play a role in diabetogenesis, and reducing systemic inflammation is often cited as a benefit of weight loss interventions.^9^

Diet may also (and independently) affect cytokine levels and inflammation.^2^ For example, the Mediterranean Diet has been shown to decrease inflammatory cytokines in many conditions.^10^ In contrast, high trans-fat diets and diets containing sugar-added foods and beverages have been shown to increase pro-inflammatory cytokines IL-6 and TNFα.^11,12^ Wheat can cause non-celiac gluten sensitivity, a hypersensitivity reaction that leads to production of IL-1β, IL-6, TNFα, and IFNγ.^13^ In contrast, fish oils containing omega-3 fatty acids decrease inflammatory cytokines IL-6 and TNFα.^14^Further, anthocyanins in blueberry and blackberry extract have been shown to reduce inflammatory cytokines induced by a high-fat diet.^15^

Despite supportive data linking diet with inflammation in several chronic diseases, diet complexity, inter-individual variability, and meals consumed as a mixture of foods rather than as isolated ingredients make it difficult to investigate the mechanisms by which an individual dietary component exerts its pro- or anti-inflammatory effects. In addition, it has been proposed that foods influence inflammation indirectly via the gastrointestinal microbiota leading to a specific inflammatory profile^16,17^ and (or) changes in gut permeability due to hypersensitivity responses to different foods affecting the cytokine production.^17,18^ The most common of these foods are citrus and nightshade vegetables, which are thought to generate sensitivities in large proportions of the population.^19,20^ Several diet-associated antigens have also been identified, although the mechanisms by which they exert their effects on the immune system have not been well-elucidated.

A variety of “anti-inflammatory” diets (also known as the hypoallergenic diet, elimination diet, and oligoantigenic diet) have been used in naturopathic medicine for the treatment of many diseases including allergies, irritable bowel syndrome, inflammatory bowel disease, rheumatoid arthritis, and systemic lupus erythematosus.^21–23^ Despite its therapeutic use, reduction in inflammation has not specifically been demonstrated with this diet.

Anti-inflammatory diets differ primarily in the recommendations regarding which foods should be excluded and included. The varying opinions amongst physicians makes it difficult to study. Nevertheless, all versions of the anti-inflammatory diet include advice regarding inclusion of fats and oils high in polyunsaturated fatty acids, particularly omega-3 fatty acids (fish, canola, flax seed, sunflower, etc.).^24,25^ In addition, limiting refined carbohydrates (white sugar, brown sugar, and honey) and emphasizing increased intake of seeds and nuts are also cornerstones of the anti-inflammatory diet, making it a low glycemic-index and glycemic-load diet. Reducing glycemic fluctuations is intended to decrease end-organ cellular oxidative stress, reactive oxygen species production, cytokine levels, and other markers of inflammation.^26–28^

This randomized controlled-feeding study addressed the question of whether the anti-inflammatory diet reduces levels of inflammatory cytokines, parameters of glucose metabolism, and cardiovascular risk factors in patients with pre-diabetes and type 2 diabetes. Participants were chosen for study because these conditions are associated with increased central obesity and levels of inflammatory markers that put them at risk for chronic diseases and poor health outcomes.^2,29^ We hypothesized that the anti-inflammatory diet would reduce inflammatory markers compared to a control diet, leading to greater improvements in glucose regulation and decreased serum lipid levels.

## METHODS

### DIET DEVELOPMENT

The anti-inflammatory diet (AI diet) used in this study was developed in collaboration with scientists and naturopathic physicians from the National University of Natural Medicine (NUNM) and scientists and bionutritionists at the Oregon Health & Science University (OHSU) Clinical and Translational Research Center (CTRC), both in Portland, Oregon. After consideration of the various recommendations regarding what constituted an anti-inflammatory diet from published sources as well as accepted practice, a group consensus was reached in which the AI diet used in this study was one that excluded foods believed to be associated with inflammation, such as wheat and other high-gluten grains, corn, soy, dairy and all dairy-containing products, nightshade vegetables (peppers, tomatoes, eggplant, potatoes), citrus, beef, pork, shellfish, eggs, trans fats, processed oils (n-6 oils), processed sugar, sugar-added foods and beverages, artificial sweeteners, caffeine, alcohol and peanuts and peanut-containing products; and included foods thought to reduce inflammation, such as those high in beneficial fatty acids and antioxidants, including fish, nuts, and darkly colored berries. The control diet was based on the American Diabetes Association (ADA) recommendations for the management of pre-diabetes and diabetes, and included ranges of macronutrient intake but no specific food restrictions.^30^

Six-day rotating meal cycles consisting of different daily menus of breakfast, lunch, dinner, and a snack were developed by the CTRC metabolic kitchen staff from original recipes (Supplemental Table 1). Nutrient composition of all foods, recipes, and meals were calculated using ProNutra diet planning software (Viocare, Princeton, NJ). Nutrient information of the foods was primarily determined using the USDA17 food database. When a matching item was not found in the database, as was the case for some specialty foods, the nutrient content of the food was resolved by the Nutrition Coordinating Center at the University of Minnesota. Additional nutrients not commonly available in the USDA17, were added from the USDA Food and Nutrient Database for Dietary Studies, 1.0 (FNDDS), Elizabeth Stewart Hands and Associates database (ESHA) (Salem, OR), and Nutrition Data System for Research (NDS) Nutrition Coordinating Center (NCC) database.

The AI and ADA diets were matched in carbohydrate, and protein (Supplemental Table 2). The AI diet met or exceeded all recommended nutrient guidelines at the 2000 kilocalorie level, with the exception of calcium and Vitamin B12. These nutrients were 55% and 92% of the DRI (dietary reference intakes), respectively. The fatty acid profile reflected a high mono and polyunsaturated fat diet, with lower than recommended saturated fat content. Cholesterol content was also far below USDA nutritional guidelines. Diet recipes and prepared meals were evaluated for palatability and acceptance during pre-study testing.

### STUDY PARTICIPANTS

**Figure 1:**
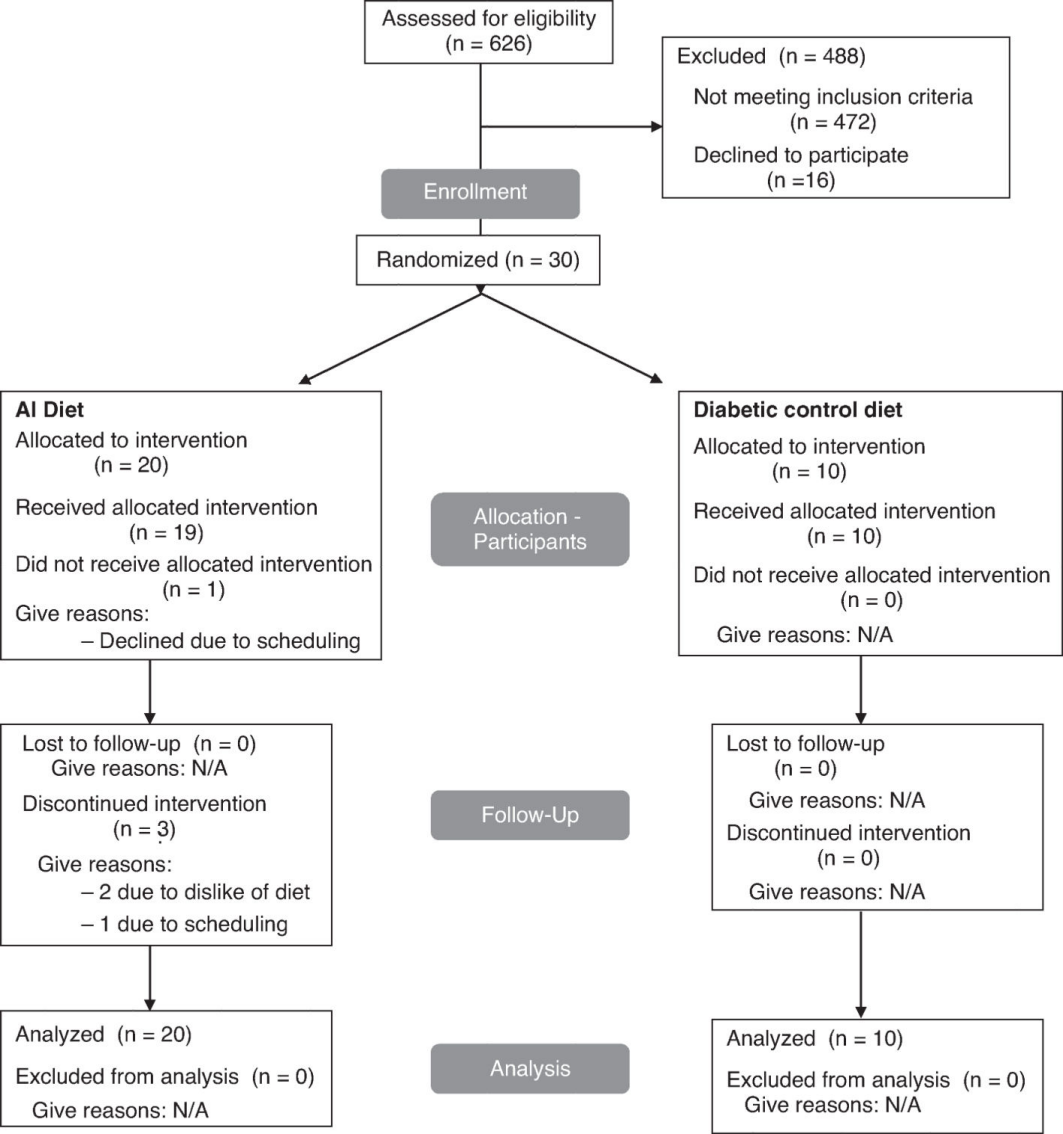
Flow diagram for study recruitment, enrollment, and retention.

Participants were recruited through advertisements in the Portland metropolitan area. Of 626 individuals contacted by phone, 104 subjects were screened in-person after giving written informed consent (Figure 1). Enrolled participants (*n*=30) who met the following criteria were included in the study: 18 to 65 years of age; BMI between 25 and 45 kg/m^2^; two fasting blood glucose measurements ≥100 mg/dL or a two-hour glucose ≥140 mg/dL obtained during an oral glucose tolerance test; no other major or chronic medical conditions; no medications or supplements that might affect inflammatory markers; not taking a medication for diabetes other than sulfonylureas; a non-smoker; and no allergies, aversions, or intolerances to foods on the study menu. The institutional review boards at both OHSU and NUNM provided ethics approval for this protocol.

### STUDY DESIGN

Participants were randomly assigned to the AI diet (*n*=20) or ADA diet (control) (*n*=10) group using a randomized, parallel-study design. This small study was designed to test recipe feasibility, diet tolerability, and cytokine variability across a six-week intervention. All study visits took place at the OHSU CTRC and were conducted between March 2006 and December 2007. Participants returned to the CTRC three times per week for breakfast and to pick-up food for the subsequent time period. During these visits, study personnel reviewed hunger and satiety scores, administered food intake questionnaires, measured body weight using a research scale at the CTRC, assessed tolerance of and consumption of the diet, discussed participants’ reported symptoms, provided encouragement, and reviewed the ongoing study schedule.

To determine the effects of each diet without confounding effects of weight loss, for the first two weeks the diets were individually calculated to provide 100% of caloric needs (isocaloric phase). The participants were instructed to eat everything provided to them, and body weight was monitored at each visit with adjustments made to the menu to maintain participant weights. To determine the effects of potential weight change resulting from dietary assignment on study outcomes, during weeks 3–6 the diets were provided in an amount estimated at 20% above isocaloric needs and participants were instructed to eat according to how hungry and full they felt *(ad libitum* phase). Following consumption, all food containers and leftover food were returned and weighed. This allowed the research team to analyze how much food was consumed.

Self-reported visual analogue scale scores of hunger and satiety were recorded. Blood draws were obtained after an overnight fast at baseline, the end of week two of the isocaloric phase, and weeks four and six of the *ad libitum* phase. A dual energy x-ray absorptiometry (DEXA) scan was performed at the beginning and end of the study to assess body composition using a Hologic QDR Discovery A Densitometer (Hologic, Inc., Bedford, MA).

### BIOLOGICAL ASSAYS

Plasma levels of pro-inflammatory cytokines TNFα, IL-1β, and IL-6 were analyzed with commercially available sandwich ELISA kits (R&D Systems, Minneapolis, MN). High-sensitivity C-reactive protein and insulin were measured using chemiluminescence-based Immulite immunoassay systems (Diagnostic Products Corporation, Los Angeles, CA). Plasma glucose concentrations were measured by calorimetric method in the CTRC Core Laboratory and whole blood hemoglobin A1c was measured by the OHSU Hospital Clinical Laboratory. The OHSU Lipid Laboratory analyzed lipoproteins using beta quantification enzymatic methods that determine total cholesterol, triglyceride levels, HDL cholesterol, and calculated VLDL cholesterol, LDL cholesterol, and non-HDL cholesterol. In samples where plasma triglyceride levels exceeded 300 mg/dL, lipoproteins were separated by preparative ultracentrifugation before analysis.

### STATISTICAL METHODS

Data on all primary variables were summarized by four time period means: at baseline, after two weeks of isocaloric feeding, midway into *ad libitum* feeding, and at the end of the study. Primary outcome measures compared across time and between groups were glucose, total cholesterol, LDL, HDL, VLDL cholesterol, triglyceride, and cytokine levels. Time-varying covariates were weight/BMI and fixed covariates of interest were age, sex, and baseline levels of the outcome measure.

An intent-to-treat analysis was used throughout. For subjects who dropped out before completion of the experiment, missing data were imputed using the last observation carried forward method. As expected, preliminary analysis indicated that the primary effect of imputation was to weaken the significance of group differences for some outcomes. For each of the primary outcome measures, we conducted three main analyses using a univariate linear mixed model ANOVA assessing the effects of diet group (as a between-subjects factor) and time period (as a within-subjects factor), especially testing for a time*group interaction. In the first primary analysis (Model 1), only baseline value of the outcome is used as a covariate. In the second (Model 2), gender is added as a covariate, in order to test whether gender moderates results. In the final analysis (Model 3), weight is added to baseline level as a time-varying covariate (without gender). Model 3 analysis tests sensitivity of results to weight change by testing for effects of diet independent of weight loss.

Hunger and satiety scores (recorded daily) were separately summarized as averages of three consecutive two-week periods and analyzed according to a single mixed model ANOVA, with diet group as a between-subjects factor and time period (first two weeks, second two weeks, last two weeks) as a within-subjects factor. Since non-spherical covariance was noted for almost all outcome measures, Greenhouse-Geisser corrections were used for repeated measures analyses. Mixed model analyses (with the weight covariate) were computed using a Huynh-Feldt covariance structure, which was determined to provide good model fit. All statistical calculations were performed using IBM SPSS v.20 (IBM Corp. Released 2011. IBM SPSS Statistics for Windows, Version 20.0. Armonk, NY: IBM Corp).

## RESULTS

Participants (53% female) were predominantly white. Mean age, gender, weight, and BMI were not statistically significant between the AI diet and the control groups at baseline (Table 1).

### DIET TOLERABILITY AND VISUAL ANALOG SCORES

Targeted dietary macronutrient and fiber intakes were achieved by study groups according to diet assignment (Supplemental Table 3). Both groups were well matched for total calorie intake during both the isocaloric and *ad libitum* feeding phases (Supplemental Table 3). Using daily visual analog scales to record the tolerability and tastiness of the diets, tolerability increased over time in both groups with no significance difference between them (data not shown).

### BODY WEIGHT AND GLUCOSE METABOLISM

During the four-week *ad libitum* feeding phase, both groups lost small but significant amounts of weight; however, change in weight was not significantly different between the two groups, according to Model 1 (Table 2).

Both dietary groups showed mean decreases in glucose levels over the course of the trial (Table 2), but there was no difference between the two groups (*P*=0.8 for interaction, Model 1); this remained true even when accounting for gender (*P*>0.5 for main effect of diet, Model 2) or weight loss (*P*=0.20 main effect of diet, Model 3).

Insulin levels showed no significant effect of diet group (*P*=0.37 for Visit*Group interaction, Model 1) (Table 2). When weight is included as a time-varying covariate, in Model 3, there continues to be no significant effect of group on changes in insulin, but we do obtain highly significant effects of visit (*P*=0.001 and weight (*P*=0.001).

### LIPID LEVELS

Total cholesterol levels decreased significantly over the course of treatment in both groups, but decreased more in the AI diet group, even when accounting for differing baseline levels between groups (Table 3).

Similarly, LDL cholesterol decreased in both groups, but the decline was greater in the AI diet group (Table 3). The significance of the effect was enhanced when weight was added as a covariate (*P*=0.017 for Visit*Group interaction, Model 3) (Table 3).

Both groups experienced reductions in HDL cholesterol over the course of treatment, but the changes over time were not significantly different between groups (*P*>0.05 for Visit*Group interaction, Model 1) (Table 3). Inclusion of gender as a factor in the model shows strong effects of gender on HDL (*P*=0.003 for gender main effect). Female subjects had higher levels of HDL in both groups, and at least in the ADA group, these fell more sharply over time than for men.

Analysis of the HDL/LDL ratio demonstrated nearsignificant differences between groups (V=0.066). Again, we observed greater differences between groups during the isocaloric phase, which slowly reversed during the *ad libitum* phase. Inclusion of gender or weight (in Models 2 and 3) did not significantly alter estimates of between-group effects.

Mean decreases in mean levels of VLDL cholesterol over time were slightly higher in the AI group (*P*=0.096) (Table 3). However, there was a significant Gender*Group*Visit interaction in Model 2. This result should be interpreted with caution, however, as it is due to two of just three male participants in the ADA group having very large increases in VLDL cholesterol over the course of the trial.

In Model 1, there was no significant effect of diet group on changes in triglyceride levels (Table 3). Similar to VLDL cholesterol, gender influenced the results on triglyceride levels as a result of three male subjects in the ADA group having very high levels at baseline that increased during the study period (*P*=0.007 for Time*Diet*Gender interaction). In Model 2, gender (*P*<0.001), group (*P*<0.001), and the Time*Group interaction (*P*=0.007) are all significant, as are the Group*Gender interaction (*P*=0.002) and the Time*Gender interaction (*P*=0.040).

### INFLAMMATORY MARKERS

Levels of TNFα, IL-6, and hs-CRP were analyzed to determine the effect of diet on inflammation (Table 4). We did not find significant evidence for effects of diet group assignment on changes in any of these markers. Participant weight had highly significant associations with both TNFα levels (*P*=0.001), and IL-6 levels (*P*=0.001) in Model 3; but inclusion of weight in the model did not result in significant effects of diet group on either outcome.

### DISCUSSION

In this pilot and feasibility study, we examined the effect of consuming an anti-inflammatory diet versus a control diet on markers of inflammation and risk factors for cardiometabolic disease. Participants were randomized to their dietary assignment under controlled feeding conditions. Each diet was matched for protein, carbohydrate, fat, and fiber content such that only the foods that made up each diet were different. In the case of the anti-inflammatory diet, several foods thought to be pro-inflammatory were eliminated and, instead, emphasis was placed on increasing intake of healthy fats^31^ and anti-inflammatory fruits and vegetables.^32^ The control diet, which was based on recommendations by the American Diabetes Association, included the foods that were eliminated as part of the AI diet, such as whole grains, red meat, nightshade vegetables, and citrus.

One of the strengths of our study was the careful matching of the two diets for nutrient content and the controlled feeding design in which all the food that was consumed was prepared by the CTRC Bionutrition staff and carefully tracked during the entire study period. This degree of precision required creativity. The AI diet is by its nature high in dietary fiber; we therefore chose a fiber amount (35–40 g/day in a 2000 kcal diet) that could be achieved in both diets in order to control for this important component. This total fiber intake is quite high compared to a typical American diet.

Because the AI diet eliminates many foods that are common in the “standard American diet,” whether the diet would be acceptable to participants was of concern. All recipes for the AI diet were taste-tested by study team members and a few test participants prior to first randomization. Further, participants were interviewed by study dieticians about their dietary preferences, so that menus could be tailored to match food preference when possible. According to visual analog scales, participants in both study groups equally enjoyed the foods to which they were assigned and felt the amount they were given was sufficient (data not shown).

Participants in both diet-treatment groups lost very modest amounts of weight and showed improvements in cardiovascular and inflammatory biomarkers over the relatively short, six-week intervention. With a few exceptions, these changes were not statistically different between the diets, including hs-CRP. The results of our study are in line with prior studies demonstrating that diets with low-glycemic index, including ones based on recommendations from the ADA, improve glycemic control in people with metabolic syndrome, pre-diabetes and type 2 diabetes.^30,33^ A 2016 meta-analysis of dietary approaches to reduce inflammation in metabolic syndrome^34^ demonstrated that low-fat diets reduced CRP compared to other diets. However, this analysis did not discriminate between types of fats in low fat diet studies. Other studies suggest that increasing healthy fats (omega-3s) is anti-inflammatory.^31,35^

Some limitations of our study deserve consideration. Relative to large epidemiological diet and uncontrolled studies, our pilot and feasibility study is relatively small (*n*=30). However, we powered our enrollment according to existing published evidence to detect differences in cytokine levels during the AI diet. We also focused our enrollment on participants known to have higher markers of inflammation (those with pre-diabetes and diabetes). Although our results were mostly not significant with regard to our primary outcomes, we can make some observations. Notably, we did find a downward trend in inflammatory cytokines levels that occurred in both diet groups during the isocaloric feeding phase and that was enhanced during the *ad libitum* phase in both diet groups when reductions in weight were seen. Therefore, we found potential benefits of both diets on inflammation, but the most significant effect is likely due to diet-independent effects of weight loss on inflammation reduction.^8,9^ An additional limitation was that participants were excluded if they reported using a variety of pharmaceuticals and supplements known or thought to affect cytokines and, in the case of those with diabetes, participants were not eligible if they took medications other than a sulfonylurea. According to consumer reports in August 2017, 55% of Americans regularly take pharmaceuticals. Therefore, our results are not generalizable to a more medicated population and future research could examine dietary effects in these groups. Finally, because there is no universally accepted “anti-inflammatory” diet, it could be argued that the dietary changes chosen for our intervention diet were incorrect or insufficient. However, we worked closely with nutritional experts in both the naturopathic and allopathic communities, and used evidence from the existing literature, to come up with a composite diet that met most guidelines for inclusion and exclusion of foods so as to optimize the body’s inflammation milieu.

## CONCLUSION

This study demonstrated that for patients with prediabetes and diabetes, both an anti-inflammatory diet and a control diet based on recommendations from the American Diabetes Association showed modest improvements in body weight and trends in benefits for inflammation and biomarkers associated with cardiovascular disease and diabetes. However, we are not able to find a specific benefit of eliminating foods that have commonly been linked with inflammation. In addition, our data suggest that any benefits in inflammation could be the result of the weight loss rather than the specific elimination of inflammatory foods and/or inclusion of anti-inflammatory fats. As a pilot and feasibility study, these conclusions should be considered preliminary and limited in generalizability until a larger trial is conducted.

## Supplementary Material

Fig. 1

Supp. data

## Figures and Tables

**Table 1:** Baseline characteristics in each diet group.

	AI group (*n*=20)		Control group (*n*=10)		*P*-value
	Mean	Std. deviation	Mean	Std. deviation	
Gender (F/M)	14/6	–	7/3	–	–
Age (years)	56.9	9.1	58.8	10.2	0.6
Weight (kg)	98.54	15.21	100.11	15.10	0.8
BMI (kg/m^2^)	33.64	3.97	33.44	4.12	0.9
Glucose (mg/dL)	113	16.3	113	9.04	>0.9
Insulin (μIU/mL)	15.83	8.86	12.32	6.60	0.3
Total cholesterol (mg/dL)	199.6	35.90	211.3	43.84	0.4
LDL cholesterol (mg/dL)	123.17	32.15	122.55	38.60	>0.9
HDL cholesterol (mg/dL)	51.25	16.66	56.1	17.64	0.5
VLDL cholesterol (mg/dL)	25.23	11.58	32.65	16.65	0.2
Triglyceride (mg/dL)	125.8	57.74	163.5	83.67	0.2
TNFα (pg/mL)	1.91	0.81	1.67	0.59	0.4
IL-6 (pg/mL)	2.43	1.32	2.89	1.02	0.4
hs-CRP (mg/dL)	4.44	3.95	3.53	2.99	0.5

AI, anti-inflammatory diet; BMI, body mass index; LDL, low density lipoprotein; HDL, high density lipoprotein; VLDL, very low density lipoprotein; TNFα, tumor necrosis factor alpha; IL-6, interleukin-6; hs-CRP, high-sensitivity C-reactive protein.

**Table 2:** Changes in parameters of weight and glucose metabolism at the end of each feeding phase compared to baseline.

	AI group			Control group		
	Baseline visit	Isocaloric end vs. baseline	*Ad libitum* end vs. baseline	Baseline visit	Isocaloric end vs. baseline	*Ad libitum* end vs. baseline
Weight (kg)						
Mean	98.6	−1.32**	−2.88***	100	−1.0*	−2.65***
Std. deviation	15.2	1.42	2.29	15.1	0.74	1.68
BMI (kg/m^2^)						
Mean	33.6	−0.45**	−0.98***	33.4	−0.31*	−0.85***
Std. deviation	3.97	0.49	0.78	4.12	0.30	0.62
Glucose (mg/dL)						
Mean	113	−4.41**	−8.19***	113**	−5.36	−8.66***
Std. deviation	16.3	6.53	9.91	9.04	3.83	6.33
Insulin (μIU/mL)						
Mean	15.8	0.84	1.62	12.3	3.89	0.59
Std. deviation	8.86	6.06	9.07	6.60	7.86	7.37

BMI, body mass index.

Significant within-group changes from baseline:

* *P*<0.05

** *P*<0.01

*** *P*<0.001.

There were no significant (*P*<0.05) between-group differences.

**Table 3:** Changes in lipid levels at the end of each feeding phase compared to baseline.

	AI group			Control group		
	Baseline visit	Isocaloric end vs. baseline	*Ad libitum* end vs. baseline	Baseline visit	Isocaloric end vs. baseline	*Ad libitum* end vs. baseline
Total cholesterol (mg/dL)						
Mean	200	−31 7***^†^	−34.6***	211	−14.6**	−27.2***
Std. deviation	35.9	23.3	25.4	43.8	14.7	15.3
LDL cholesterol (mg/dL)						
Mean	123	−24 1***^†^	−24.83***	123	−9.95*	−20.7***
Std. deviation	32.2	18.6	19.9	38.6	14.94	13.96
HDL cholesterol (mg/dL)						
Mean	51.3	−3.85***	−5.78***	56.1	−7.4***	−8.8***
Std. deviation	16.66	4.89	5.46	17.64	7.11	8.19
VLDL cholesterol (mg/dL)						
Mean	25.2	−3.68**^†^	−4.0**^†^	32.7	2.75	2.25
Std. deviation	11.6	5.54	6.98	16.7	5.20	13.7
Triglycerides (mg/dL)						
Mean	126	−18.4**^†^	−20.0**^†^	164	13.5	10.7
Std. deviation	57.7	28.0	35.0	83.7	26.2	68.0

LDL, low density lipoprotein; HDL, high density lipoprotein; VLDL, very low density lipoprotein.

Significant within-group changes from baseline:

* *P*<0.05

** *P*<0.01

*** *P*<0.001.

Significant difference in changes from baseline between groups:

† *P*<0.05.

**Table 4:** Changes in markers of inflammation at the end of each feeding phase compared to baseline.

	AI group			Control group		
	Baseline visit	Isocaloric end vs. baseline	*Ad libitum* end vs. baseline	Baseline visit	Isocaloric end vs. baseline	*Ad libitum* end vs. baseline
TNFα (pg/mL)						
Mean	1.91	−0.17†	−0.12	1.67	0.07	−0.01
Std. deviation	0.81	0.37	0.42	0.59	0.17	0.19
IL-6 (pg/mL)						
Mean	2.43	−0.12	−0.12	2.89	−0.39	−0.28
Std. deviation	1.32	0.59	0.74	1.02	0.74	1.11
hs-CRP (mg/dL)						
Mean	4.44	−1.18	−1.70	3.53	−0.69	−0.12
Std. deviation	3.95	3.55	3.55	2.99	1.14	2.30

IL-6, interleukin-6; TNFα, tumor necrosis factor alpha; hs-CRP, highly-sensitive C-reactive protein. There were no significant (*P*<0.05) within-group changes. Significant difference in changes from baseline between groups:

† *P*<0.05.
