# Supplementary figures and images for: Effect of the Anti-Inflammatory Diet in People with Diabetes and Pre-Diabetes: A Randomized Controlled Feeding Study
Source: J Restor Med. Author manuscript; Available in PMC 2019 Jun 5. (PMC6550471; doi:10.14200/jrm.2019.0107)

**Figure 1. Anti-Inflammatory Diet Flow Diagram**

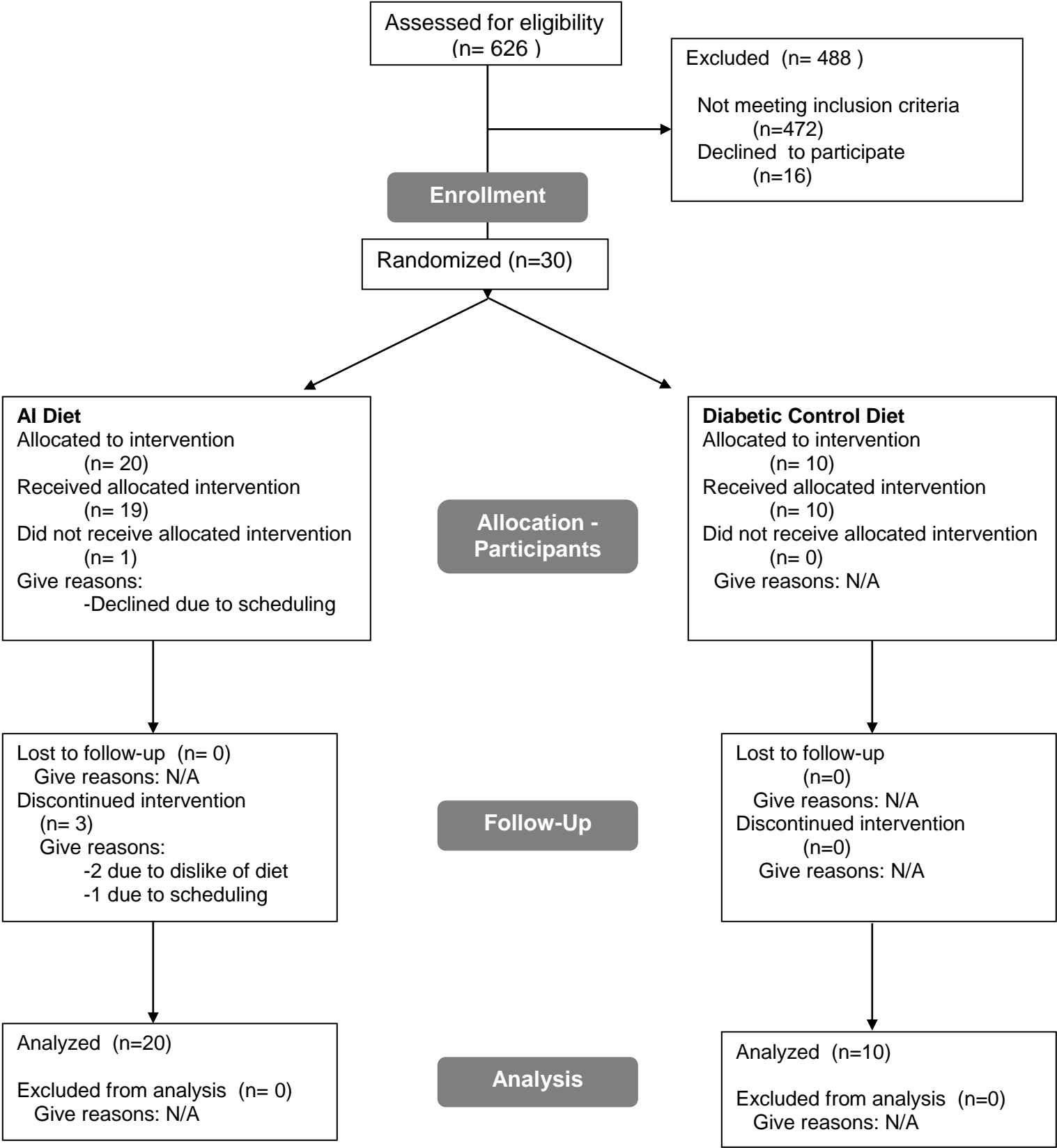

Supplement: Fig. 1 [file NIHMS1020614-supplement-Fig__1.pdf]
