# Supplementary material for: Effect of the Anti-Inflammatory Diet in People with Diabetes and Pre-Diabetes: A Randomized Controlled Feeding Study
Source: J Restor Med. Author manuscript; Available in PMC 2019 Jun 5. (PMC6550471; doi:10.14200/jrm.2019.0107)
Supplement: Supp. data [file NIHMS1020614-supplement-Supp__data.pdf]

Supplemental Table 1. Sample menus for participants assigned to the anti-inflammatory and control diets.

Anti-inflammatory Diet Menu

	Day 1	Day 2	Day 3	Day 4	Day 5	Day 6
Breakfast	Yam latkes with warm cinnamon applesauce and walnuts<br>Apple chicken sausage<br>Guava juice<br>Ground flaxseed	Millet rice cereal with dried Blueberries and hazelnut milk<br>Pecan raisin bread with almond butter<br>Ground flaxseed	Blueberry pancakes with agave<br>Apple chicken sausage<br>Apple<br>Rice milk<br>Ground flaxseed	Hot quinoa breakfast cereal<br>Pecan bread with almond butter<br>Mangos<br>Rice milk<br>Ground flaxseed	Fruit and nut hot cereal<br>Almonds<br>Rice milk<br>Pineapple<br>Ground flaxseed	Berry smoothie<br>Pecan bread with cashew butter and all fruit jam<br>Ground flaxseed
Lunch	Chicken salad sandwich with leaf lettuce on pecan bread<br>Pumpkin curry soup<br>Pears	Turkey sandwich with iceberg lettuce, cucumber, mock mayonnaise and mustard on millet bread<br>Red lentil dal<br>Peaches<br>Almonds	Almond sesame noodles<br>Romaine and spinach salad with balsamic vinaigrette	Tuna/white bean salad<br>Rice tortilla<br>Romaine and spinach salad with walnuts and ginger dressing<br>Dried apricots	Baked chicken<br>Gingered butternut squash soup<br>Romaine and spinach salad with cucumber, garbanzo beans and balsamic vinaigrette<br>Sweet potato chips<br>Pears	Salmon and vegetable pasta<br>Romaine and spinach salad with beets, carrots and tahini dressing<br>Hummus<br>Rice crackers
Dinner	Baked halibut<br>Mango rice pilaf<br>Peas & carrots<br>Romaine and spinach salad with ginger dressing<br>Millet bread and olive oil	Baked chicken<br>Quinoa veggie salad<br>Wilted spinach and almonds<br>Pear crisp	Ginger salmon<br>Herbed mushroom rice<br>Broccoli<br>Mashed sweet potatoes<br>Cashew almond cookies	French lentils over brown rice<br>Wilted spinach and almonds<br>Blueberry crisp	Fresh herb halibut<br>Mixed grains<br>Coleslaw	Chicken stir fry<br>Brown rice<br>Cashews<br>Peaches
Snack	Nut & seed granola<br>Frozen blueberries<br>Hazelnut milk	Omega-3 bar<br>Carrots and tahini dressing	CLIF nectar bar<br>Pineapple	Black olives<br>Celery<br>Cashews	Lara bar<br>Broccoli with tahini dressing	Brown rice cereal bars<br>Apple

Control Diet Menu

	Day 1	Day 2	Day 3	Day 4	Day 5	Day 6
Breakfast	Eggo waffles with margarine and<br>light pancake syrup<br>Walnuts and dried apricots<br>Hard-boiled egg<br>Skim Milk	Cheerios<br>English muffin with peanut butter and jam<br>Skim milk	Corn meal pancakes with margarine and light pancake syrup<br>Yogurt with frozen raspberries and pistachios	Quaker granola<br>Yogurt<br>Whole wheat toast with margarine<br>Skim milk<br>Grapefruit	Spinach potato frittata<br>Whole wheat toast with margarine and jam<br>Canned pears<br>Skim milk	Whole wheat bagel with cream cheese and jam<br>Yogurt<br>Peaches

Lunch	Bean soup and cornbread<br>Mixed lettuce salad with tomato and cucumber<br>Salt-free turkey<br>Mozzarella cheese stick<br>Fat-free Italian dressing<br>Oat maple cookie	Tofu pate sandwich on whole wheat bread with iceberg lettuce and tomato<br>Minestrone soup<br>Orange<br>Pretzels	Ham sandwich on whole wheat bread with cheddar cheese, mustard, mayonnaise, iceberg lettuce and tomatoes<br>Roasted red pepper & tomato soup with saltines<br>Skim milk	Chicken salad on whole wheat pita bread with tomato<br>Mixed lettuce salad<br>Italian dressing<br>Mixed peanuts, raisins and Cheerios	Corn and zucchini chowder<br>Mixed lettuce salad with tomatoes, red peppers and light ranch dressing<br>Ritz crackers<br>Cheddar cheese	Roast beef sandwich on whole wheat bread with cheese, tomato and mayonnaise<br>Mixed lettuce salad with carrots and fat-free thousand Island dressing<br>Baked kettle chips
Dinner	Bowties w/ spinach, beef casserole<br>Dinner roll with margarine<br>Peas and carrots<br>Skim Milk	Beans and rice with chicken breast<br>Iceberg lettuce salad with tomatoes and red peppers<br>Ranch dressing<br>Canned peaches<br>Graham crackers	Tenderloin steak<br>Mashed potatoes<br>Garbanzo bean salad<br>Broccoli<br>Jello (sugar-free)<br>Pineapple	Spaghetti with marinara veggie sauce and parmesan cheese<br>Wheat roll (refined wheat) with margarine<br>Whole kernel corn<br>Vanilla wafers<br>Skim milk	Pork tenderloin<br>Scallion rice<br>Mandarin oranges<br>Broccoli<br>Whole wheat dinner roll with margarine	Vegetable & chicken stir fry<br>Soba noodles<br>Salt-free peanuts<br>Jello (sugar-free)<br>Skim milk
Snack	Salt-free peanuts<br>Canned pears	Saltine crackers<br>Cheddar cheese<br>Chocolate pudding	Apple<br>Oats and honey granola bar	Tortilla chips<br>Salsa<br>Dried apricots	Peanut butter<br>Graham crackers<br>Skim milk	Luna bar<br>Pistachios<br>Apple

Supplemental Table 2. Targeted nutrient content of intervention and control diets.

Nutrient	Anti-inflammatory Diet	Control Diet
Protein (% kcal)	15%	18%
Carbohydrate (% kcal)	50%	51%
Fat-Total (% kcal)	35%	31%
Saturated (% kcal)	5%	9%
Poly (% kcal)	11%	8%
Mono (% kcal)	18%	12%
Cholesterol (mg)	84	153
Fiber (g)	35	28

Supplemental Table 3. Actual macronutrient and fiber intake according to diet group assignment.

Nutrient	Anti-inflammatory Diet		Control Diet	
	Isocaloric Phase (Weeks 1-2)	Ad libitum Phase (Weeks 3-6)	Isocaloric Phase (Weeks 1-2)	Ad libitum Phase (Weeks 3-6)
Total calories (kcal)	2785	2332	2804	2427
Total fat (g)	113	96	99	86
% kcal from fat	37%	37%	32%	32%
Total carbohydrates (g)	357	293	371	317
% kcal from carbohydrates	51%	50%	53%	52%
Protein (g)	109	93	129	114
% kcal from protein	16%	16%	18%	19%
Fiber (g)	47	38	39	33

Data are average intakes during the study period indicated as determined by CTRC Bionutrition staff from supplied and returned food.
